# A Case Series on a Layered Biomaterial Strategy for Midface Rejuvenation: Combining Collagen Stimulators and Hyaluronic Acid

**DOI:** 10.1111/jocd.70815

**Published:** 2026-03-23

**Authors:** Yin‐Jie Ao, Ying‐Jin Zhou

**Affiliations:** ^1^ Plastic Surgery and Medical Cosmetic Center, The Affiliated Eye Hospital of Jiangxi Medical College Nanchang University Nanchang China

## Abstract

**Background:**

The midface is a key area where aging signs first appear, involving multi‐layered changes such as epidermal fine lines, volume loss leading to hollowing and sagging, and deep structural support weakening resulting in nasolabial fold deepening. Single‐material treatments often fail to address this complexity comprehensively. While hyaluronic acid (HA) effectively restores volume, its impact on skin texture is limited; collagen stimulators promote long‐term collagen regeneration but lack immediate shaping capability.

**Objective:**

Through the presentation of six typical cases, this study aims to illustrate the clinical application of a layered treatment strategy using HA and collagen stimulators, either alone or in combination. It seeks to describe how this anatomy‐based approach can address single or compound midface aging signs and provide a preliminary clinical reference for comprehensive rejuvenation.

**Methods:**

This descriptive case series included six female patients with distinct midface aging phenotypes. Efficacy was assessed blindly by independent physicians using standardized scales (GAIS, Merz, Hirmand, MVDSS, WSRS).

**Results:**

All cases showed improvement in target aging signs. Quantitative improvements were observed: Case 3 (infraorbital hollowing) improved from Merz Grade 2 to 1; Case 4 from Merz Grade 4 to 1; Case 5 (tear trough) improved from Hirmand Grade 2 to 0 at 6 months; and Case 6 (compound aging) showed progressive improvement, with Hirmand, MVDSS, and WSRS scores all improving by at least two grades at the 10‐month follow‐up. Treatments were well‐tolerated, with only transient, expected reactions observed.

**Conclusion:**

This case series suggests that a combined/layered treatment strategy based on anatomical presentation may offer a safe and effective option for personalized midface rejuvenation. These preliminary observations provide a basis for future research and should be interpreted within the limitations of a small, non‐comparative study design.

## 1 Introduction

The midface represents a focal point in facial aging, where the earliest and most perceptible signs of senescence emerge [1]. Anatomically, aging in this region unfolds across multiple tiers; the epidermis and dermis undergo thinning and textural change due to collagen and hyaluronic acid (HA) depletion, presenting as fine lines and loss of luminosity; the subcutaneous soft tissue compartments suffer from volume attenuation and gravitational descent, leading to infraorbital hollowing, malar fat pad ptosis, and tear trough formation; and the deep structural foundation—comprising retaining ligaments and bony architecture—weakens over time, accentuating nasolabial fold depth and midface flattening [2, 3, 4]. This multidimensional, layered progression poses a significant clinical challenge, as monotherapeutic approaches often prove insufficient to address the full spectrum of aging manifestations [5, 6, 7].

Currently, two principal classes of injectable agents are employed in midface rejuvenation: HA and collagen stimulators. HA fillers excel in providing immediate volume restoration and structural repositioning, yet their capacity to improve skin quality and texture remains limited. Conversely, collagen stimulators promote neocollagenesis and gradual tissue restoration, offering sustained improvements in skin elasticity and mild volumetric correction, but they lack the immediate lifting and contouring effect of fillers [8]. Given these complementary mechanisms—where HA addresses the “space” and collagen stimulators target the “structure”—a rational, layer‐adapted combination of both modalities may yield synergistic benefits, enabling more holistic and durable rejuvenation outcomes [9].

Accordingly, given the complementary mechanisms of HA and collagen stimulators, a combination approach is theoretically attractive. However, clinical evidence on how to optimally combine these modalities based on individual aging phenotypes remains limited. To address this gap, we present this descriptive case series to share our preliminary experience with a structured, anatomy‐based treatment framework. Through six representative cases, we aim to illustrate the decision‐making process and clinical outcomes of this layered approach, providing a reference for clinicians and generating hypotheses for future investigations.

## 2 Materials and Methods

### 2.1 Case Selection

#### 2.1.1 Rationale for Case Selection

The cases in this study were derived from the first author's clinical practice between March 2024 and June 2025. During this period, all patients receiving midface injection treatments were preliminarily assessed; however, only those patients who exhibited typical characteristics consistent with one of the five predefined anatomical‐clinical phenotypes and had complete follow‐up data were included in this series for analysis. This purposive sampling strategy was designed to maximize the representativeness and educational value of each aging phenotype.

#### 2.1.2 Inclusion and Exclusion Criteria and Rationale for Case Selection

##### 2.1.2.1 Inclusion Criteria

Age range: 25–50 years old (adult to middle‐aged women).

Primary Aging Signs: Presence of at least one clearly defined, patient‐identified midface aging sign with a positive desire for treatment.

Representativeness: The presenting aging sign must be clearly classifiable into a typical anatomical‐clinical subtype of midface aging.

##### 2.1.2.2 Exclusion Criteria

Pregnancy or lactation.

Active facial infection, inflammatory skin disease, or unhealed wounds in the treatment area.

Uncontrolled systemic diseases (e.g., severe diabetes, autoimmune disorders) or coagulation disorders.

Known allergy to HA, collagen stimulators, or any component of their formulations.

History of permanent filler implantation in the treatment area or presence of scar tissue that would interfere with assessment.

This study included only female patients aged 25–50 years, which limits the generalizability of our findings to male patients and to individuals outside this age range. Midface aging patterns and treatment responses may differ across sexes and age groups. Future studies should include more diverse populations to validate the applicability of this layered treatment strategy in broader clinical settings.

*Case 1*: Epidermal and superficial dermal aging.

*Represented Problem*: Fine lines and wrinkles, stemming from dermal matrix (collagen, HA) degradation leading to textural deterioration.

*Case 2*: Dermal‐subcutaneous junction pigmentation and microcirculation issues.

*Represented Problem*: Pigmented dark circles involve factors like dermal pigmentation, subcutaneous vascular prominence, and soft tissue thinning.

*Cases 3 and 4*: Subcutaneous and submuscular soft tissue volume loss.

*Represented problem*: Infraorbital hollowing.

Case 3 (Physiological) represents primary, age‐related fat pad atrophy.

Case 4 (Iatrogenic) represents secondary, structural fat deficiency following surgery, a common sequela in East Asian populations.

*Case 5*: Multi‐layer composite defect (ligamentous laxity with mild volume deficiency).

*Represented Problem*: Tear trough deformity, with a pathological basis combining deep tear trough ligament laxity and superficial SOOF fat pad atrophy.

*Case 6*: Full‐layer, multidimensional composite aging.

*Represented Problem*: Pan‐facial aging involving skin, fat, ligaments, and bony support, manifesting as multiple signs including tear trough, malar fat pad descent, and nasolabial fold deepening.

### 2.2 Materials

The following materials were utilized in the corresponding cases:

1. For fine lines, wrinkles, and dark circles. NCTF 135 (FILLMED Laboratories, France), a sterile solution containing HA, vitamins, antioxidants, amino acids, and coenzymes, was employed for intradermal or subdermal injection.
2. For infraorbital hollowing and sub‐orbicularis oculi volumization. Polycaprolactone (PCL)—based collagen stimulator (Purajuve, Shandong Guyuchun Biotechnology Co. Ltd., China) was used after dilution with sterile normal saline.
3. For deep structural support and volume restoration. HA dermal filler (FILLMED UNIVERSAL, FILLMED Laboratories, France) was applied in the periosteal or deep fat compartment.
4. For large‐area skin tightening and collagen stimulation. Poly‐L‐lactic acid (PLLA) injectable (PULIYAN, Puliya (Nanjing) Medical Technology Co. Ltd., China) was administered subdermally after reconstitution.

All products are approved medical devices in China. The selection of specific products for each case was based on their rheological properties and the clinician's judgment of the most suitable material for the target tissue layer. All patients were fully informed about the nature of the products used, including any off‐label applications (such as specific dilution protocols for collagen stimulators) and provided written informed consent before treatment.

### 2.3 Treatment Strategy and Technique

**FIGURE 1:**
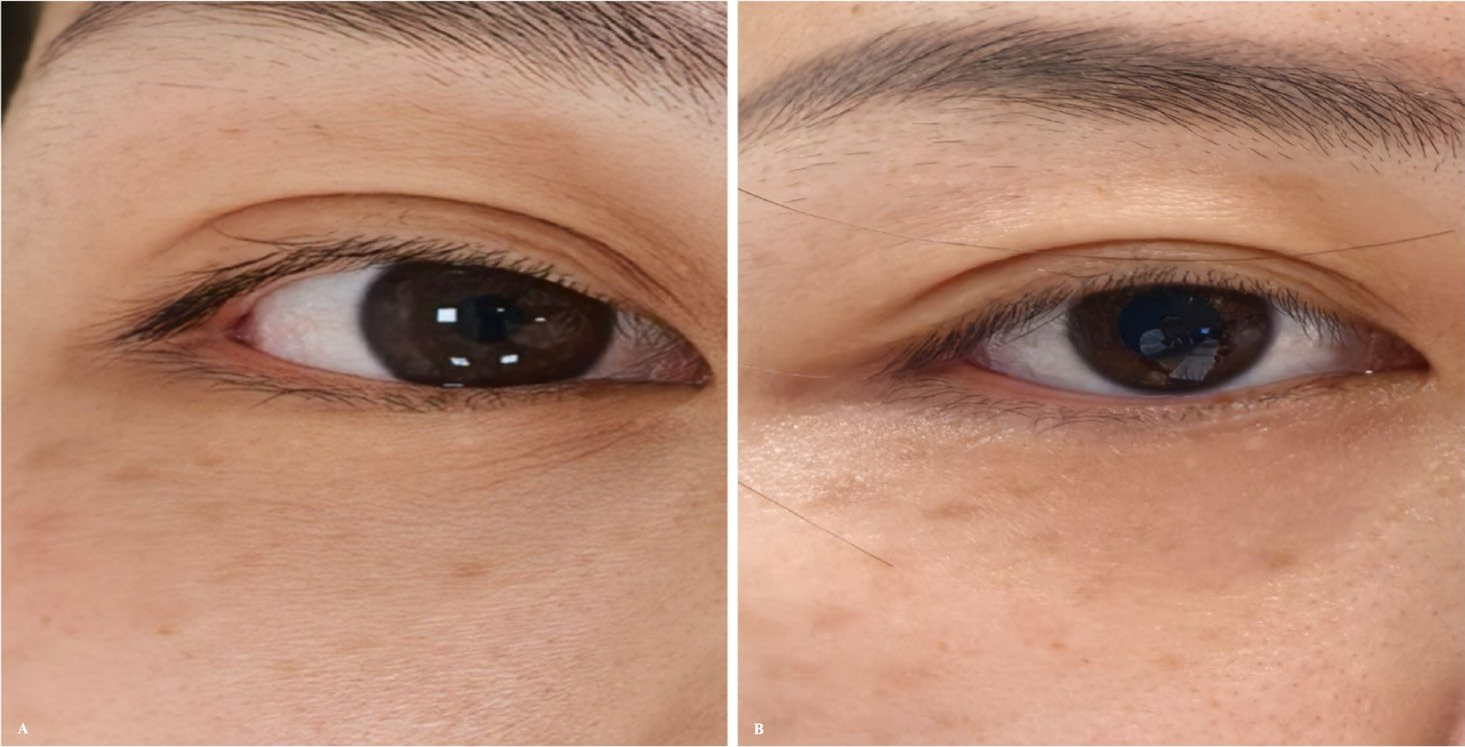
(A) Pre‐treatment baseline. (B) Three‐month follow‐up.

*Case 1*: Epidermal and superficial dermal aging—A 34‐year‐old female presented with infraorbital fine lines and wrinkles. She was otherwise healthy with no prior history of cosmetic or surgical procedures (Figure 1, Table 1).

**TABLE 1:** Etiological analysis and injection details/strategy for Case 1.

Represented problem	Material	Level and gauge and injection technique	Dosage
Fine lines and wrinkles	NCTF 135	Intradermal/32G/Needle	0.8 mL per side
GAIS: 3 month versus preoperation	+1		

**FIGURE 2:**
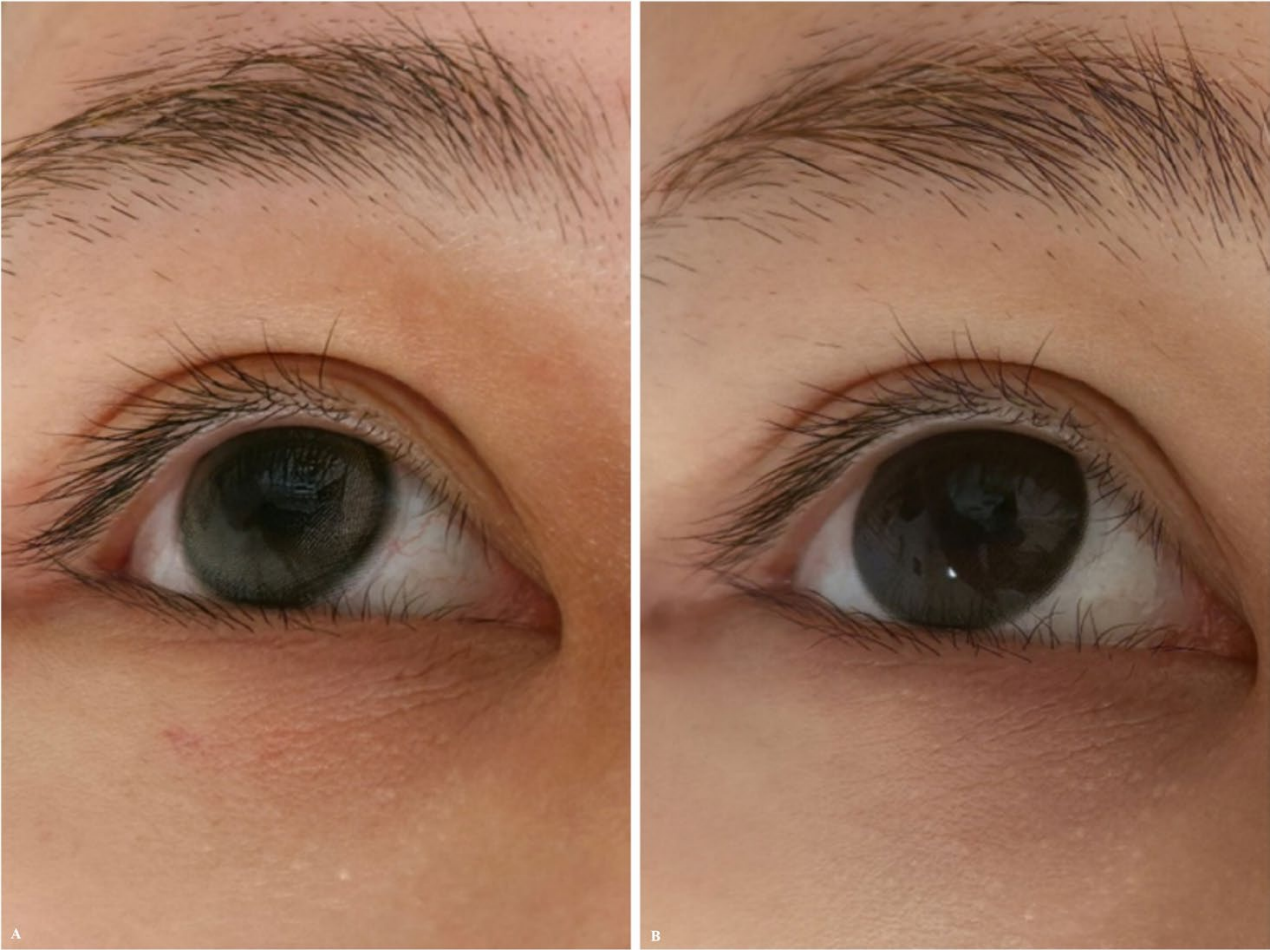
(A) Pre‐treatment baseline. (B) Three‐month follow‐up.

*Case 2*: Dermal‐Subcutaneous Junction Pigmentation and Microcirculation Issues—A 42‐year‐old female presented with pigmented dark circles. She was otherwise healthy with no prior history of cosmetic or surgical procedures (Figure 2, Table 2).

**TABLE 2:** Etiological analysis and injection details/strategy for Case 2.

Represented problem	Material	Level and gauge and injection technique	Dosage
Pigmented dark circles	NCTF 135	Intradermal/27G/Cannula	2.5 mL per side
GAIS: 3 month versus preoperation	+1		

*Cases 3 and 4*: Subcutaneous and submuscular soft tissue volume loss.

Case 3 (Physiological) represents primary, age‐related fat pad atrophy.

**FIGURE 3:**
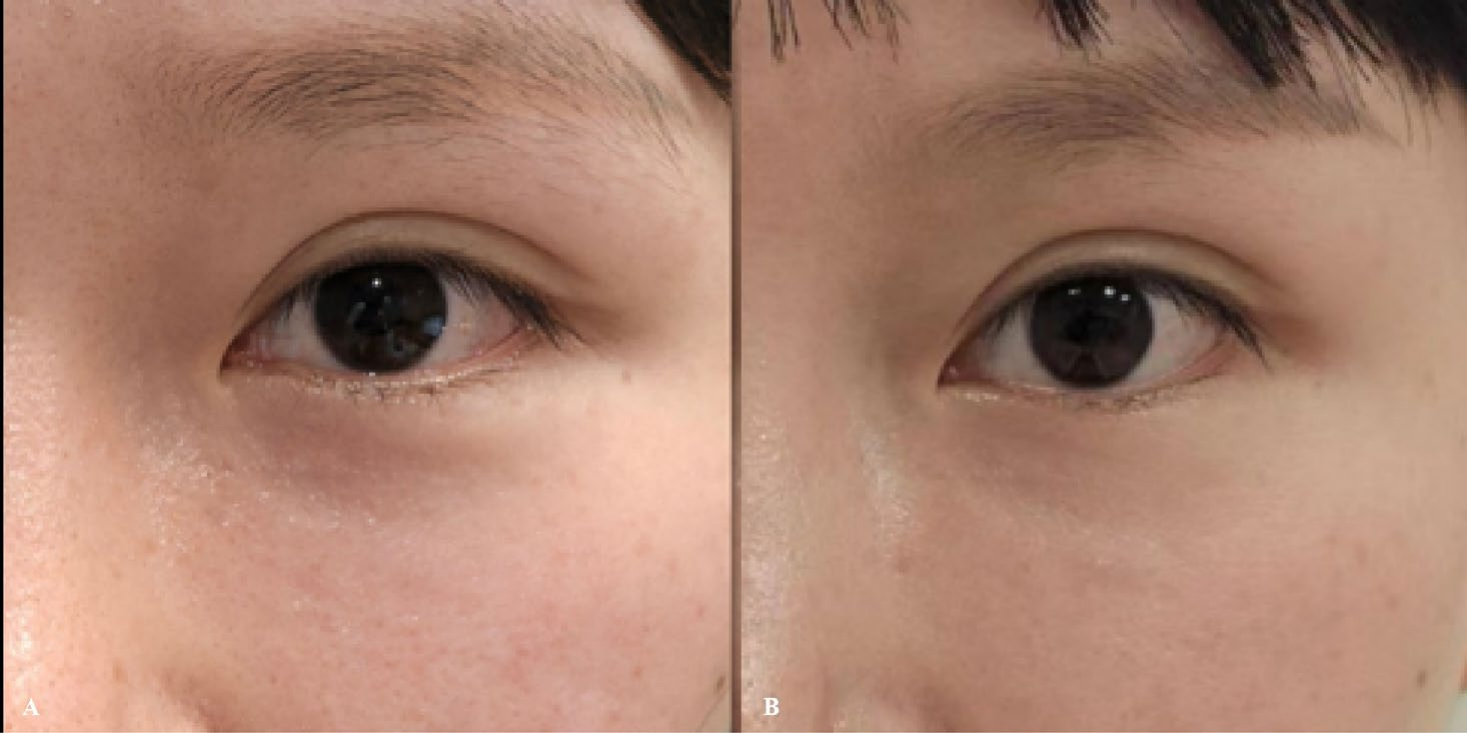
(A) Pre‐treatment baseline. (B) Three‐month follow‐up.

**FIGURE 4:**
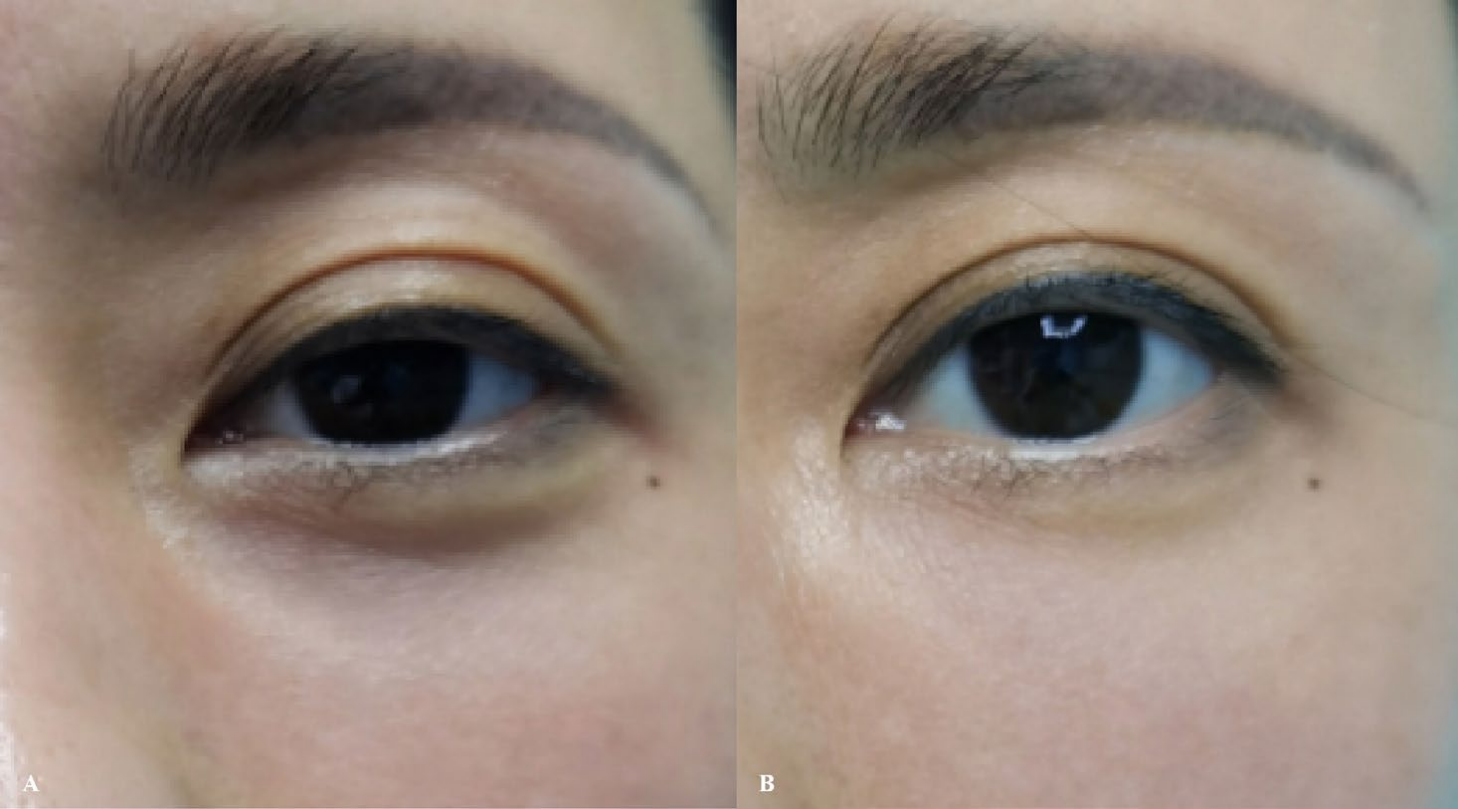
(A) Pre‐treatment baseline. (B) Three‐month follow‐up.

Case 4 (Iatrogenic) represents secondary, structural fat deficiency following surgery, a common sequela in East Asian populations (Figures 3 and 4, Table 3).

**TABLE 3:** Etiological analysis and injection details/strategy for Cases 3 and 4.

Represented problem	Material	Level and gauge and injection technique	Dosage
Infraorbital hollowing			
Case 3: Congenital	PCL + 0.5 mL NS	Sub‐orbicularis oculi/27G/Cannula	0.2 mL per side
Merz: 3 month versus preoperation	2–1		
Case 4: Acquired	PCL + 0.5 mL NS	Sub‐orbicularis oculi/27G/Cannula	0.4 mL per side
Merz: 3 month versus preoperation	4–1		

**FIGURE 5:**
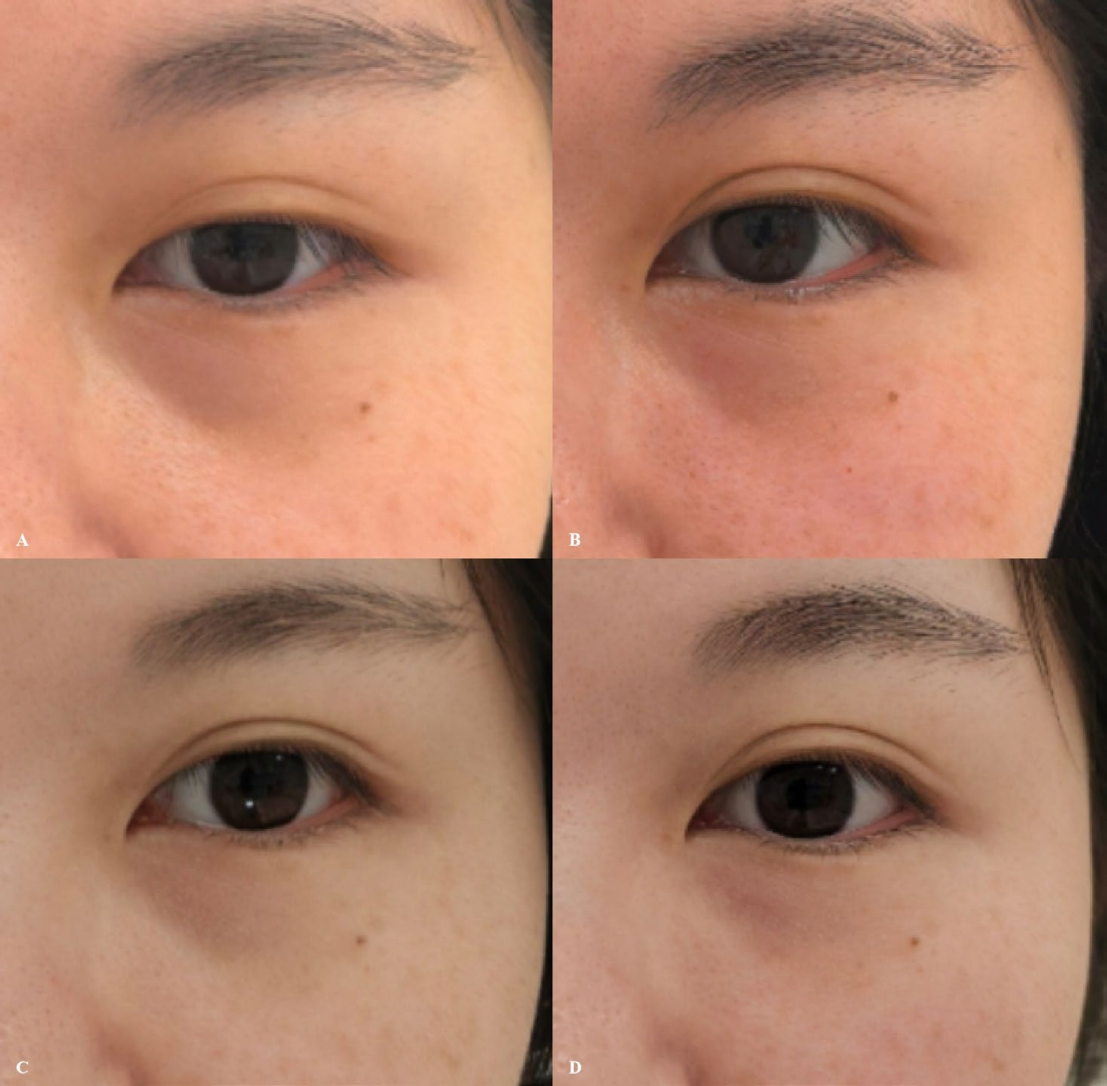
(A) Preoperative baseline. (B) Immediate postoperative appearance. (C) Appearance at 3‐month follow‐up. (D) Appearance at 6‐month follow‐up.

*Case 5*: Multi‐layer composite defect (ligamentous laxity with mild volume deficiency) (Figure 5, Table 4).

**TABLE 4:** Etiological analysis and injection details/strategy for Case 5.

Represented problem	Material	Level and gauge and injection technique	Dosage
Tear trough deformity	AFU	Periosteum/23G/Needle	0.1 mL per side
	PCL + 0.5 mL NS	Sub‐orbicularis oculi/27G/Cannula	0.2 mL per side
Hirmand:preoperation versus 3 month versus 6 month		2–1–0	

**FIGURE 6:**
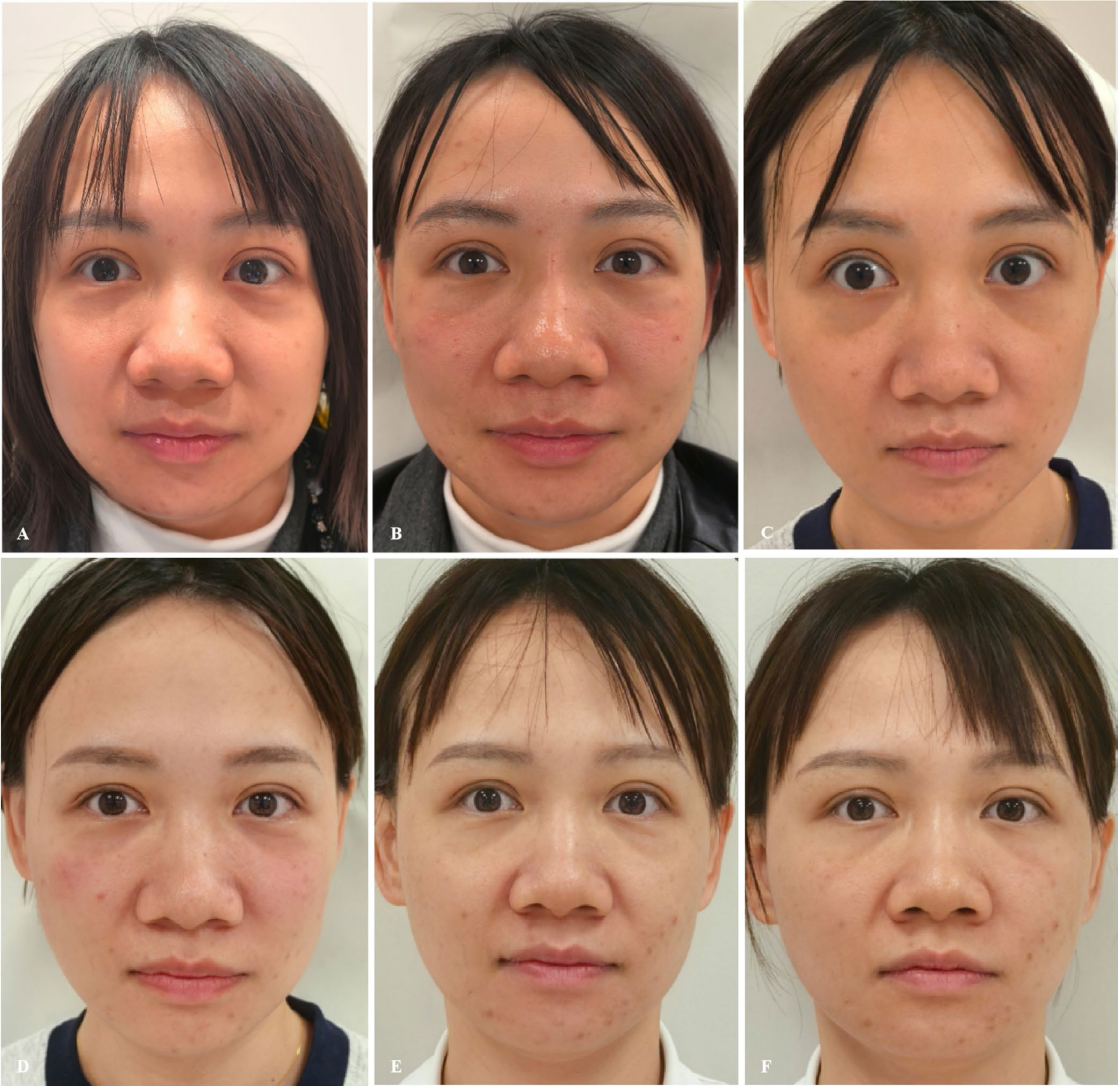
(A) Preoperative baseline. (B) Immediate postoperative appearance. (C) Appearance at preoperative assessment of the 3‐month follow‐up session. (D) Appearance immediately after retreatment at the 3‐month follow‐up session. (E) Appearance at preoperative assessment of the 10‐month follow‐up session. (F) Appearance immediately after retreatment at the 10‐month follow‐up session.

*Case 6*: full‐layer, multidimensional composite aging (Figure 6, Table 5).

**TABLE 5:** Etiological analysis and injection details/strategy for Case 6.

Represented problem	Material	Level and gauge and injection technique	Dosage
Tear trough, malar fat pad descent, and nasolabial fold deepening	AFU	Periosteum/23G/Needle	0.2 mL per side
	PCL + 0.5 mL NS	Sub‐orbicularis oculi/27G/Cannula	0.4 mL per side
	PLLA + 6 mL NS	Subcutaneous/27G/Cannula	3 mL per side
Supplementary injection at 3 months	AFU	Periosteum/23G/Needle	0.2 mL per side
	PCL + 0.5 mL NS	Sub‐orbicularis oculi/27G/Cannula	0.4 mL per side
Supplementary injection at 10 months	AFU	Periosteum/23G/Needle	0.2 mL per side
Efficacy evaluation	Preoperation	3 months	10 months
Hirmand	3	2	1
MVDSS	3	2	1
WSRS	4	3	2
GAIS		+1	+2

Primary treatment effect: Improvements observed within 3–6 months after the initial injection. Maintenance treatment effect: Further improvements achieved through retreatment based on the initial results.

### 2.4 Evaluation and Follow‐Up

Evaluation methods. Standardized photography, clinical rating scales.

Follow‐up time points. Determined based on the specific treatment protocol, commonly at 3, 6, and 10 months post‐treatment [10].

Evaluation methods and rating systems.

Global esthetic Improvement Scale (GAIS). −2: Much Worse, −1: Worse, 0: No Change, +1: Improved, +2: Much Improved [11].

Merz Infraorbital Hollow Assessment Scale. Grade 0: None to Minimal, Grade 1: Mild, Grade 2: Moderate, Grade 3: Severe, Grade 4: Extreme [12].

Midface Volume Deficit Severity Scale (MVDSS). Grade 0: None/Minimal, Grade 1: Mild, Grade 2: Moderate, Grade 3: Severe, Grade 4: Very Severe [13].

Hirmand Tear Trough Deformity Severity Scale (HIRMAND). Grade 1: None/Mild, Grade 2: Moderate, Grade 3: Severe [14].

Wrinkle Severity Rating Scale (WSRS). Grade 1: No wrinkles, Grade 2: Shallow wrinkles, Grade 3: Moderately deep wrinkles, Grade 4: Deep wrinkles, well‐defined folds, Grade 5: Very deep wrinkles, redundant folds [15].

Efficacy assessment was performed independently and blindly by a physician not involved in the treatment. Prior to evaluation, all pre‐ and post‐treatment photographs were randomized and time information was concealed, so that evaluators could not identify the treatment stage corresponding to each image. The two evaluators scored independently, and the results were averaged; if the score difference exceeded one grade, consensus was reached through discussion.

## 3 Results

### 3.1 Case 1

Immediate post‐treatment swelling around the eyes and slight bruising at some needle puncture sites were observed, both resolving spontaneously within 72 h, with no other discomfort reported. At the 3‐month follow‐up, a reduction in fine lines and dryness of the lower eyelid was noted. Compared with the pre‐treatment condition, a GAIS score of 1 (Improved) was recorded (Figure 1).

### 3.2 Case 2

Immediate post‐treatment swelling around the eyes and slight bruising along the blunt cannula tracks were noted, resolving spontaneously within 72 h, with no other discomfort. At the 3‐month follow‐up, pigmented dark circles were visibly lightened. A GAIS score of 1 (Improved) was given compared to the pre‐treatment state (Figure 2).

### 3.3 Case 3

Mild periorbital swelling occurred immediately after treatment and resolved within 72 h, with no other discomfort reported. At the 3‐month follow‐up, infraorbital hollowing was improved. In cases of physiological infraorbital hollowing, treatment resulted in an improvement in the Merz score from 2 (*moderate*) to 1 (*mild*) (Figure 3).

### 3.4 Case 4

Immediate post‐treatment periorbital swelling was observed and resolved within 72 h, with no other discomfort. At the 3‐month follow‐up, significant improvement in infraorbital hollowing was noted. In cases of iatrogenic infraorbital hollowing, treatment led to a significant improvement in the Merz score from 4 (*extreme*) to 1 (*mild*) (Figure 4).

### 3.5 Case 5

Post‐treatment periorbital swelling occurred and resolved within 72 h, with no other discomfort. At the 3‐month follow‐up, tear trough improvement was observed, with the Hirmand score decreasing from 2 to 1. At the 6‐month follow‐up, the Hirmand score further improved to 0 (Figure 5).

### 3.6 Case 6

Immediate post‐treatment swelling of the periorbital area and lateral face was noted, resolving within 72 h without other discomfort.

Three‐month follow‐up after initial treatment. Tear trough improvement, Hirmand score decreased from 3 to 2. Improvement in malar fat pad ptosis, MVDSS score decreased from 3 to 2. Nasolabial fold improvement, WSRS score decreased from 4 to 3. Overall improvement, GAIS score was 1 (Improved; Figure 6).

Comparison between pre‐treatment, 10‐month follow‐up, and immediate post‐injection at 10 months. Tear trough continued to improve, Hirmand score decreased progressively from 3 to 2 to 1. Malar fat pad ptosis continued to improve, MVDSS score decreased progressively from 3 to 2 to 1. Nasolabial folds continued to improve, WSRS score decreased progressively from 4 to 3 to 2. Significant overall improvement: GAIS score was 2 (Much Improved; Figure 6).

## 4 Discussion

This case series describes our preliminary experience with an anatomy‐based, layered treatment strategy combining HA and collagen stimulators for midface rejuvenation. Across six representative cases, we observed that matching material properties—HA for immediate structural support and collagen stimulators for progressive tissue regeneration to specific anatomical layers and aging phenotypes yielded favorable clinical outcomes.

The evolution of injectable fillers provides important context for understanding the rationale behind combination approaches. Fat grafting, once a mainstream option, remains limited by unpredictable survival rates (reported in the literature to range between 20% and 80%) and variability in outcomes due to differences in processing techniques and physician experience [16, 17]. These limitations have contributed to the increasing preference for synthetic fillers in contemporary esthetic practice, particularly when predictability and precision are prioritized. HA fillers have been widely adopted for their immediate volumizing effect and favorable safety profile [18]. However, the esthetic medicine literature has increasingly recognized that inappropriate injection techniques—particularly overzealous volumization—can lead to suboptimal outcomes. The term “facial overfilling syndrome” has been used in peer‐reviewed publications to describe these sequelae [19]. Notably, some industry marketing has promoted “regenerative materials” as a solution to the “dough‐like” appearance sometimes associated with HA overfilling [15].

A balanced examination of the literature suggests that such characterizations may oversimplify a complex issue. Commercially available collagen stimulators can be categorized into two types: those without immediate support (e.g., traditional PLLA) and those with immediate support provided by a carrier gel (e.g., composite CaHA or PCL products) [20]. Studies have shown that as a microsphere carrier, HA gel may offer advantages over carboxymethyl cellulose (CMC) gel in terms of tissue regeneration and volume stability [16]. This literature‐based evidence suggests that the “dough‐like” appearance sometimes observed with HA fillers is more likely attributable to injection depth, dosage, product selection, and technical execution rather than to HA itself [18].

Thus, the emergence of collagen stimulators should be viewed not as a replacement for HA but as a complementary tool that, when combined with HA in a layer‐adapted manner, may expand the clinician's ability to address the multidimensional nature of facial aging. This contextual understanding informed the treatment approach presented in our case series.

In Cases 1 and 2, superficial injection of a HA‐based nutrient complex improved fine lines and pigmented dark circles, respectively, suggesting that intradermal or subdermal delivery of such formulations may benefit epidermal and superficial dermal aging. In Cases 3 and 4, sub‐orbicularis oculi injection of PCL effectively corrected both physiological and iatrogenic infraorbital hollowing, with Merz scores improving from 2 to 1 and from 4 to 1, respectively. These observations support the use of PCL as a volumizing agent in the deep infraorbital compartment, consistent with previous reports on collagen stimulators for periorbital rejuvenation [21, 22]. Case 5 demonstrated that combining periosteal HA with submuscular PCL can address the multi‐layer defect underlying tear trough deformity—HA providing immediate ligamentous support and PCL contributing to progressive soft tissue regeneration. The Hirmand score improved from 2 to 0 over 6 months, with continued improvement beyond the initial effect of HA, suggesting a synergistic benefit of the combined approach. This finding aligns with the conceptual framework described in the literature that HA addresses “space” while collagen stimulators target “structure.” [5] Case 6, representing full‐layer multidimensional aging, received a combination of HA, PCL, and PLLA across multiple anatomical planes. Progressive improvement was observed across all assessed parameters (Hirmand, MVDSS, WSRS) over 10 months, with GAIS scores improving from +1 at 3 months to +2 at 10 months following a touch‐up treatment. While the use of multiple products in a single case precludes isolation of individual material contributions—a limitation we acknowledge—the cumulative outcome illustrates the potential of a comprehensive, layer‐adapted strategy for complex aging presentations.

Across all six cases, only transient and expected post‐treatment reactions were observed, including mild swelling and occasional bruising, all resolving spontaneously within 72 h. No cases of nodule formation, granuloma, or other delayed complications were reported during the follow‐up period (up to 10 months). These findings are consistent with the established safety profiles of HA and collagen stimulators when used appropriately, as documented in the literature [23, 24]. Regarding the comparative safety of these material classes, the literature indicates that HA exhibits high biocompatibility with minimal inflammatory response and predictable metabolism [23]. Collagen stimulators, by contrast, function through a controlled foreign‐body reaction that stimulates collagen production—a mechanism that inherently involves a degree of inflammation. According to published studies, large‐volume injection or bolus deposition of microspheres may increase the risk of medium‐ to long‐term granuloma formation, suggesting these materials may be less suitable for substantial volumization compared to HA [25]. These literature‐based observations informed our treatment protocols, in which HA was used for structural volumizing while collagen stimulators were placed in appropriate planes at appropriate dilutions to optimize safety. The absence of granuloma or nodule formation in our cases, while reassuring, should be interpreted with caution given the small sample size and limited follow‐up duration—a point emphasized in our limitations section.

The concept of combining HA and collagen stimulators is supported by a growing body of literature [9]. In a systematic review of combined and hybrid treatments, reported that such approaches may offer synergistic benefits by leveraging the immediate volumizing effect of HA and the sustained biostimulatory effect of collagen stimulators [10]. Lorenc et al. demonstrated that a combined PLLA and HA regimen enhanced facial harmony and skin quality [10]. Our observations in Cases 5 and 6 are consistent with these reports, suggesting that a layered, anatomy‐based combination strategy may be particularly valuable for patients with multi‐layer aging involvement. Regarding layer‐specific material selection, the literature indicates that cross‐linked HA is generally not recommended for superficial periorbital areas due to the risk of visible lumps or the Tyndall effect, while appropriately diluted collagen stimulators can be used cautiously in superficial layers to improve skin texture and mild volume deficits [24, 26]. Our approach in Cases 1–4 adhered to these principles, with superficial injection of nutrient solution for fine lines and dark circles, and deeper placement of PCL for volumizing indications.

## 5 Limitations

This study has several limitations: First, methodological limitations. As an exploratory case series, it lacks a control group and cannot quantitatively evaluate the independent effects of each material or exclude natural recovery or placebo effects. Second, selection bias. Cases were deliberately selected rather than consecutively enrolled to illustrate typical applications of the layered strategy. While consistent with case series methodology, this introduces selection bias, limiting generalizability. Third, sample limitations. The small sample size (six cases) and inclusion of only females aged 25–50 years, though reflecting the mainstream esthetic population, limit generalizability to males, other ages, and ethnic groups. Fourth, limited follow‐up. With a maximum follow‐up period of only 10 months, this study is insufficient to assess long‐term durability and safety, particularly for collagen stimulators with progressive mechanisms. The limited duration also precludes the evaluation of rare delayed complications such as granulomas or late‐onset nodule formation. Future studies with larger sample sizes and extended follow‐up are therefore necessary to comprehensively validate the long‐term safety of this treatment protocol. Fifth, procedural variability. Use of multiple products and case‐specific injection techniques reflects real‐world individualized treatment but limits reproducibility. Sixth, this study relied on clinician‐rated scales without incorporating validated patient‐reported outcome measures such as the FACE‐Q questionnaire. Future studies should include standardized patient‐reported instruments to capture the full spectrum of treatment benefits. In summary, these findings represent preliminary observations. Future research should include larger, prospective controlled trials with extended follow‐up, objective assessments, and diverse populations. Seventh, although photographs were taken under consistent conditions, we did not use laboratory‐grade standardization equipment such as head fixation devices or constant‐color temperature lighting. While we implemented standardized protocols (fixed equipment, consistent environment, uniform patient positioning, and blinded evaluation), the lack of fully controlled photographic conditions remains a methodological limitation that may affect the objectivity of visual assessments. Eighth, this retrospective study was conducted before ultrasound guidance was routinely available in our practice; therefore, real‐time imaging verification of product placement at target anatomical layers could not be provided. This represents a methodological limitation.

Despite its limitations, this case series offers several clinically relevant insights. First, it demonstrates that a structured, anatomy‐based approach to material selection and injection planning is feasible in routine practice. Second, it suggests that combining HA and collagen stimulators may be particularly beneficial for patients with multi‐layer aging involvement, where monotherapy would be insufficient. Third, it provides preliminary evidence that PCL can effectively correct both physiological and iatrogenic infraorbital hollowing when placed in the sub‐orbicularis plane. Fourth, the progressive improvement observed in Cases 5 and 6 suggests that collagen stimulators may contribute to ongoing tissue regeneration beyond the initial effect of HA, potentially extending treatment durability.

## 6 Conclusion

This case series describes our preliminary experience with an anatomy‐based, layered treatment strategy combining HA and collagen stimulators for midface rejuvenation. Across six representative cases, we observed that matching material properties—HA for immediate structural support and collagen stimulators for progressive tissue regeneration—to specific anatomical layers and aging phenotypes yielded favorable clinical outcomes. These observations suggest that a combined approach may offer advantages over monotherapy in addressing the multidimensional nature of midface aging, particularly when aging signs involve multiple tissue layers.

However, these findings should be interpreted as hypothesis‐generating rather than conclusive. As an uncontrolled case series with a small sample size, this study cannot establish the superiority of combined therapy, nor can it support definitive clinical recommendations. The optimal selection and sequencing of materials, as well as the comparative efficacy of different combination strategies, remain important questions for future investigation.

Nevertheless, this layered framework provides a useful conceptual model for clinical decision‐making and may serve as a foundation for designing larger, prospective studies. Future research with controlled designs, validated patient‐reported outcomes, and longer follow‐up is needed to validate these preliminary observations and refine treatment algorithms for personalized midface rejuvenation.

## Author Contributions

**Yin‐Jie Ao:** contributed to study design and conceptualization, main manuscript writing and revision, manuscript submission, and performed all treatment procedures and data documentation for the cases. **Ying‐Jin Zhou:** responsible for patient follow‐up, efficacy data collection and organization, and standardized archiving of imaging materials.

## Funding

The authors have nothing to report.

## Ethics Statement

This study was approved by the Affiliated Eye Hospital of Nanchang University Ethics Committee, with approval number YLP20240439. All treatments and data collection procedures were conducted in accordance with the principles of the Declaration of Helsinki, and informed consent was obtained from all patients.

## Consent

All patients included in this case series provided written informed consent for the treatment procedures and for the use of their clinical data and photographs for research and educational purposes. Written consent has been obtained regarding the publication of identifiable images (including eyes and periorbital regions) in an online open‐access journal, with the understanding that every effort has been made to minimize identifying features while maintaining scientific integrity. Patients were assured that their personal information would be handled confidentially and that they could withdraw consent at any time without affecting their future medical care.

## Conflicts of Interest

The authors declare no conflicts of interest.

## Data Availability

All data generated or analyzed during this study are included in this published article. The clinical photographs and evaluation scores are presented within the manuscript and its tables/figures.

## References

[1] P. Molinari , S. U. Urso , C. Faso , I. Iacovone , and G. Mosti , “Mastering Midface Rejuvenation: A Proposal for a New Three‐Layer, Ultrasound‐Guided Technique,” Journal of Cosmetic Dermatology 25, no. 1 (2026): e70664. 41532951 10.1111/jocd.70664 PMC12802819

[2] J. Y. Park , D. J. Im , H. D. Jeon , et al., “A Split Face Study Comparing the Effect of a PDLLA Based Product and PLLA on the Nasolabial Fold (NLF),” Skin Research and Technology 32, no. 1 (2026): e70324. 41532837 10.1111/srt.70324 PMC12802405

[3] D. Shome , S. Parkar , R. Shahare , et al., “Revolutionizing Tear Trough Rejuvenation: A Novel Bilaminar Approach With Differential Rheologic G Prime Fillers,” Indian Journal of Ophthalmology 74, no. 1 (2026): 133–140. 41460143 10.4103/IJO.IJO_2934_24 PMC12867301

[4] K. K. Durairaj , M. Yambao , J. Linnemann‐Heath , and A. Dhiman , “Sculpting the Midface and Lower Face: A Novel Biostimulatory Technique Using Hyperdilute Calcium Hydroxylapatite,” Aesthetic Surgery Journal Open Forum 7 (2025): ojaf104. 41376672 10.1093/asjof/ojaf104 PMC12686807

[5] N. Fakih‐Gomez and J. Kadouch , “Combining Calcium Hydroxylapatite and Hyaluronic Acid Fillers for Aesthetic Indications: Efficacy of an Innovative Hybrid Filler,” Aesthetic Plastic Surgery 46, no. 1 (2022): 373–381. 34341855 10.1007/s00266-021-02479-x PMC8831259

[6] R. Meçani , M. Amiri , J. Kadouch , et al., “Combined and Hybrid Treatments of Hyaluronic Acid (HA) and Calcium Hydroxylapatite (CaHA): A Systematic Review of Mechanisms of Action, Aesthetic Effectiveness, Satisfaction, and Safety Profile,” Aesthetic Plastic Surgery 49, no. 19 (2025): 5292–5313. 40481158 10.1007/s00266-025-04904-x PMC12594701

[7] Z. P. Lorenc , M. Somenek , T. Q. Nguyen , et al., “A Multicenter, Open‐Label Study of Combined Poly‐L‐Lactic Acid and Hyaluronic Midface Filler Regimen Enhances Facial Harmony and Skin Quality in GLP‐1 Medication Users,” Aesthetic Surgery Journal 12, no. 2 (2025): 374–381. 10.1093/asj/sjaf240 PMC13064655 41243519

[8] M. Aggarwal , P. Ghosh , K. P. Mohithraj , et al., “Hydrothermal Synthesis of Light‐Responsive Adhesive Protein Polymeric Dot Nanogel for Wound Healing,” ACS Applied Materials & Interfaces 23, no. 1 (2026): 73–81. 10.1021/acsami.5c18059 41610514

[9] V. Siramangkhalanon , “Visual Aesthetics (VA) Methodology: A Strategic Approach to Facial Rejuvenation,” Journal of Cosmetic Dermatology 24, no. 12 (2025): e70593. 41395849 10.1111/jocd.70593 PMC12704028

[10] C. F. Sollitto , M. Narduzzi , and C. Wolinsky , “A Systematic Review of Platelet‐Rich Plasma Versus Platelet‐Rich Fibrin for Periorbital Rejuvenation,” Journal of Cosmetic Dermatology 24, no. 11 (2025): e70524. 41190633 10.1111/jocd.70524 PMC12587466

[11] H. E. Lee , J. Wan , J. K. Song , et al., “Hybrid Poly‐D,L‐Lactic Acid and Autologous Fat Transfer for Forehead Augmentation,” Aesthetic Plastic Surgery 15, no. 1 (2026): 143–154. 10.1007/s00266-026-05622-8 41588091

[12] C. Puyana and J. R. Montes , “Long‐Term Effects of Tear Trough Hyaluronic Acid Filler: A Retrospective Study,” Journal of Clinical and Aesthetic Dermatology 18, no. 11 (2025): 44–47. PMC12724988 41446717

[13] B. Rzany , M. Sulovsky , G. Sattler , M. Cecerle , and D. Grablowitz , “Long‐Term Performance and Safety of Princess VOLUME PLUS Lidocaine for Midface Augmentation: The PRIMAvera Clinical Study,” Aesthetic Surgery Journal 44, no. 2 (2024): 203–215. 37439274 10.1093/asj/sjad230 PMC10790962

[14] C. Wang , C. Wu , and H. Liang , “Assessment of the Efficacy of Tear Trough Depression Reconstruction Using an Intraoral Cannula‐Guided Puncture for Orbital Septum Fat Flap With the FACE‐Q Scale,” Journal of Craniofacial Surgery 12, no. 1 (2025): 144–154. 10.1097/SCS.0000000000012070 41100203

[15] M. D. Maruichi , T. C. Rodrigues , A. F. Yamada , A. Skaf , and I. R. B. Godoy , “MRI Findings of Aesthetic Gluteoplasty‐From Pre‐ to Postoperative: A Review and Pictorial Essay,” Skeletal Radiology 16, no. 1 (2026): 43–54. 10.1007/s00256-026-05142-1 41611900

[16] D. Castelanich , L. A. Parra , A. M. Amado , et al., “Enzymatic Management of Facial Overfilled Syndrome: A Case Series and Narrative Review,” Journal of Cosmetic Dermatology 24, no. 8 (2025): e70377. 40781937 10.1111/jocd.70377 PMC12334978

[17] X. Liu , Y. Hu , H. Liang , S. Feng , Y. Wei , and D. Huang , “Aldehyde‐Mediated Carboxymethylcellulose/Gelatin‐Based Injectable Dual Network Hydrogel With Antimicrobial and Adhesion Properties for Rapid Hemostasis and Wound Repair,” Carbohydrate Polymers 376 (2026): 124817. 41611431 10.1016/j.carbpol.2025.124817

[18] Z. Liao , X. Chen , R. Brusini , et al., “Non‐Crosslinked Hyaluronic Acid Redensity 1() Supports Cell Viability, Proliferation, and Collagen Deposition in Early Burn Management,” Pharmaceutics 18, no. 1 (2025): 143–154. 10.3390/pharmaceutics18010021 PMC12845186 41599128

[19] C. X. Peng , W. Xv , and Y. J. Ao , “A Review: Causes, Consequences, and Management Strategies of Facial Overfilling,” Clinical, Cosmetic and Investigational Dermatology 18 (2025): 1857–1864. 40785833 10.2147/CCID.S539888 PMC12333868

[20] K. Goldie , G. Ferland‐Caron , V. Vachiramon , B. Viscomi , and S. Sattler , “Universal Lip Integrity, Proportion, and Structure: A Framework for Achieving Consistent, Natural‐Looking Lip Augmentation Using Hyaluronic Acid Fillers,” Aesthetic Surgery Journal Open Forum 7 (2025): ojaf131. 41608464 10.1093/asjof/ojaf131 PMC12836120

[21] M. Cavallini , A. Braz , D. Greiner‐Krüger , et al., “Global Recommendations for Facial Rejuvenation Using a Hyaluronic Acid and Calcium Hydroxyapatite Hybrid Injectable,” Journal of Cosmetic Dermatology 25, no. 1 (2026): e70608. 41566538 10.1111/jocd.70608 PMC12824049

[22] F. Khorasanizadeh , A. Momeni , A. Daneshvar , R. Ghannadzadeh , I. Etesami , and X. Wortsman , “Ultrasonographic Evaluation of Cosmetic Fillers: Patterns and Frequent Complications—A Literature Review,” Clinical Imaging 131 (2026): 110708. 41505983 10.1016/j.clinimag.2025.110708

[23] N. Hessloehl , P. Y. Collart‐Dutilleul , O. Romieu , and D. Carayon , “Functional and Aesthetic Oral Rehabilitation After Cancer Treatment Using Dental Prosthesis and Hyaluronic Acid Injections: A Case Report and Review of Literature,” World Journal of Clinical Cases 14, no. 2 (2026): 110627. 41608147 10.12998/wjcc.v14.i2.110627 PMC12836028

[24] L. N. Trinh , K. C. McGuigan , and A. Gupta , “Delayed Complications Following Dermal Filler for Tear Trough Augmentation: A Systematic Review,” Facial Plastic Surgery 38, no. 3 (2022): 250–259. 34666405 10.1055/s-0041-1736390

[25] X. Wortsman , Y. Valderrama , G. Ortiz‐Orellana , et al., “International Multicentric Study on Ultrasound Characteristics, Layer Location, and Corporal Distribution of Granulomas After Cosmetic Fillers Injections,” Journal of Ultrasound in Medicine 44, no. 8 (2025): 1447–1455. 40186407 10.1002/jum.16700

[26] J. Cheng , G. Bao , D. Lin , et al., “Silk Fibroin Counteracts Fibroblast Senescence to Restore ECM Homeostasis in Aged Skin,” Bioactive Materials 58 (2026): 666–684. 41551197 10.1016/j.bioactmat.2025.12.006 PMC12808896

